# Cuban epidemic neuropathy: lessons learned from scientific and political controversies

**DOI:** 10.3389/fphar.2026.1927625

**Published:** 2026-10-14

**Authors:** Alina González-Quevedo, Rosaralis Santiesteban Freixas, Francisco H. Figaredo Curiel

**Affiliations:** 1 Instituto de Neurología y Neurocirugía, Havana, Cuba; 2 Centro de Estudios Demográficos de la Universidad de La Habana, Havana, Cuba

**Keywords:** Cuban epidemic neuropathy, mitochondrial dysfunction, optic neuropathy, social determinants of health, toxic–nutritional neuropathy, vitamin deficiency

## Abstract

**Introduction:**

Cuban epidemic neuropathy (CEN), which emerged in the early 1990s during Cuba’s Special Period, was one of the largest neurological epidemics of the 20th century and became the subject of a scientific and political controversy.

**Objective:**

To revisit the epidemic and examine the lessons learned from the scientific debates, institutional responses, and political context that shaped its investigation and management.

**Development:**

This perspective integrates epidemiological, clinical, neuropathological, therapeutic and health evidence, to analyze competing etiological hypotheses and the influence of geopolitical and institutional factors on scientific interpretation. Accumulated evidence—including severe dietary restriction, depletion of B-group vitamins and other nutrients, increased metabolic demands, tobacco and alcohol exposure, and axonal pathology—supports a multifactorial toxic–nutritional model, over a viral agent. Nutritional depletion may have reduced mitochondrial metabolic reserve, antioxidant and detoxification capacity, and increased oxidative stress. Mitochondrial dysfunction and oxidative injury provide a convergent mechanism for selective vulnerability of the optic nerve and peripheral axons. Therapeutic implications underscore the benefit of early nutritional intervention even when the specific contributions of individual nutrients or toxins remain uncertain. The scientific controversy illustrates how geopolitical and institutional factors influenced the interpretation of science, research priorities, communication, and public health decision-making.

**Conclusion:**

CEN demonstrates the importance of multidisciplinary evidence, scientific independence, transparent communication, early intervention, and recognition of social determinants in neurological epidemics. These lessons remain highly relevant as Cuba today confronts renewed economic hardship and disturbing geopolitical challenges in progress.

## 1 Introduction

The Cuban epidemic of optic and peripheral neuropathy in the early 1990s was one of the largest neurological epidemics of the 20th century, ultimately affecting more than 50,000 people nationwide by January 1994. It emerged in the midst of a profound socioeconomic disruption in Cuba known as the Special Period, which followed the collapse of the Soviet Union and the socialist bloc between 1989 and 1991 and the tightening of the United States blockade. The abrupt interruption of longstanding trade relationships and external supplies produced severe shortages of fuel, food, fertilizers, medicines, and other essential goods, with major consequences for nutrition, physical activity, healthcare, and living conditions (Centers for Disease Control and Prevention, 1994; Román, 1994a; Garfield and Santana, 1997; Vayssière, 2000).

The magnitude of this disruption was reflected in a marked contraction of the Cuban economy. By 1992, Gross Domestic Product (GDP) had fallen by approximately 35% compared with 1989, while the availability of calories and protein declined substantially and dietary diversity was reduced (Vayssière, 2000; Figaredo Curiel et al., 2023). Fuel shortages disrupted electricity generation and transportation and increased the physical demands imposed on the population. At the same time, restricted access to international markets complicated the procurement of medicines, vitamins, diagnostic supplies, and research materials. The collapse of the socialist trading system and the tightening of US restrictions represented interacting, but historically distinct, determinants of this crisis (Centers for Disease Control and Prevention, 1994; Román, 1994a; Garfield and Santana, 1997; Vayssière, 2000; Román, 1998; Figaredo Curiel et al., 2023). Thus, the epidemic emerged within a setting in which nutritional, metabolic, environmental, and socioeconomic stressors converged at an unprecedented scale.

The first cases, recognized in late 1991 in the Western tobacco-growing province of Pinar del Rio, initially presented predominantly as an optic disorder. Most of those patients were middle-aged men, frequently smokers and alcohol consumers, who developed subacute bilateral visual impairment, reduced color discrimination, and central or centrocecal scotomas. The optic fundus was often normal at onset or showed only subtle temporal pallor, though subsequent loss of fibers in the papillomacular bundle became evident. As the epidemic progressed, a predominantly peripheral form was increasingly recognized, particularly among women, characterized by sensory symptoms, paresthesia, and weakness. Myelopathy and hearing impairment were reported less frequently. These manifestations were later classified into optic, peripheral, and mixed optic–peripheral forms, reflecting the spectrum of a broader neurological syndrome that came to be known as Cuban epidemic neuropathy (CEN) (Llanos et al., 1993; Román, 1994b; Sadun et al., 1994).

The rapid rise in cases and their geographic spread generated an urgent need not only to identify the cause but also to determine whether the disease could be treated or prevented. The resemblance of the optic syndrome to tobacco–alcohol amblyopia, together with its occurrence during severe nutritional deprivation, led several Cuban clinicians and researchers to propose a toxic–nutritional mechanism early in the investigation. Nevertheless, other toxic, infectious, and inflammatory contributors were also investigated, including cerebrospinal fluid (CSF) abnormalities, peripheral inflammatory biomarkers, and potential exposure to fertilizers and pesticides, although these possibilities were subsequently discarded (Colectivo de Autores, 1995).

This interpretation immediately suggested a practical intervention: if impaired nutritional and metabolic reserve played a role, then nutritional repletion could potentially both improve affected patients and prevent new cases. At that point, however, reports of viral isolation from the CSF in a high percentage of patients reinforced the possibility of an infectious origin, and the confrontation between the toxic–nutritional and viral hypotheses reached its peak (Sadun et al., 1994). By March 1993, the epidemic had affected over 45,000 individuals, prompting the establishment of a National Task Force and, in May 1993, the implementation of a nationwide multivitamin supplementation program (Centers for Disease Control and Prevention, 1994; Colectivo de Autores, 1995; Román, 1995).

Given the scale and unusual clinical characteristics of the epidemic, Cuba sought international assistance. Following a formal request at the 46th World Health Assembly on 4 May 1993, the Pan American Health Organization (PAHO) assembled a multidisciplinary mission including specialists in nutrition, neurotoxicology, neuro-ophthalmology, neurology, epidemiology, and virology. The PAHO team arrived in Havana on 16 May 1993, together with a team organized by ORBIS, a nonprofit organization based in New York dedicated to the global fight against blindness. Their objectives included reviewing the clinical and epidemiological evidence, evaluating competing etiological hypotheses, supporting diagnostic and research efforts, and assessing appropriate therapeutic and preventive measures (Román, 2016; González-Quevedo et al., 2018).

International collaboration subsequently expanded the scientific investigation of CEN. The 1994 International Workshop on Cuban Epidemic Neuropathy in Havana marked an important stage in this process. The evidence presented supported the interpretation of CEN as a toxic–metabolic axonal neuropathy associated primarily with the profound reduction in nutritional intake, rather than as an infectious or inflammatory disease. Scientists from the United States and other countries subsequently contributed to detailed analyses of the epidemic and its possible mechanisms (Hedges et al., 1997).

However, the scientific investigation unfolded within a highly charged political environment. CEN became the focus of competing interpretations in which nutritional deprivation, toxic exposures, infectious agents, and external factors were assigned different degrees of importance. These interpretations were inseparable from the broader ideological tensions of the post–Cold War period and influenced scientific communication, research priorities, therapeutic priorities, international collaboration, and public narratives surrounding the epidemic (Figaredo Curiel et al., 2023).

More than 3 decades later, CEN remains a unique natural experiment for understanding how nutritional, metabolic, toxic, and environmental stressors can converge on vulnerable neural systems. This article revisits CEN from a wider perspective, focusing on three interconnected dimensions: the scientific evidence underlying its toxic–nutritional and metabolic interpretation; the development of therapeutic and preventive interventions and their impact at a population-level; and the political and institutional circumstances that shaped the scientific debate.

## 2 Etiological debate: scientific evidence and political context

The etiological debate surrounding CEN largely centered on two competing hypotheses: toxic–nutritional and viral etiology. Both were explored in parallel during the early phases of the epidemic.

Robust epidemiological, clinical, biochemical, and therapeutic evidence supported the toxic–nutritional hypothesis during the first year of the outbreak (1992). The main findings of the preliminary investigations were initially documented in official reports of the Ministry of Public Health and subsequently published. These included the severity and abruptness of food shortages, lower risk for developing the disease in individuals with a higher intake of animal nutrients and B group vitamins; and the high prevalence and magnitude of rapid weight loss among affected individuals - average per person weight loss in the general population over the year previous to the outbreak was 10–20 lb. Additionally, widespread changes in dietary and lifestyle patterns, led to a striking shift toward a diet with a lower intake of protein, fat, vitamins, and minerals and a higher intake of carbohydrate, together with an increase of physical exercise due to fuel shortages in the midst of high environmental temperatures (Llanos et al., 1993; Hedges et al., 1997; The Cuba Neuropathy Field Investigation Team, 1995). There was evidence of markedly lower intakes of energy, proteins - especially of animal origin—essential amino acids, methionine, fats, B-group vitamins (mainly B12, riboflavin, niacin, folate and pyridoxine), antioxidant carotenoids and tobacco and alcohol use with a higher risk of disease (Centers for Disease Control and Prevention, 1994; Gay et al., 1995; Porrata et al., 1995; Jiménez et al., 1995). Furthermore, biochemical studies demonstrated thiamine depletion in a substantial proportion of the general population, particularly in regions with higher disease incidence (Macías-Matos et al., 1996). These observations were complemented by clinical evidence of relatively rapid improvement in optic and neuropathic symptoms following intensive parenteral and oral multivitamin therapy during 10 days, followed by oral administration for up to 6 months. Depending on the severity and other concurring comorbidities, recovery was observed in approximately 75% of patients at 21 days and in 95% at 1 year. However, the prognosis was poor if treatment was delayed more than 4 months after disease onset (Mojon et al., 1997; Santiesteban-Freixas et al., 2010).

Taken together, the consistency of the epidemiological associations, objective biochemical evidence of micronutrient depletion, characteristic dietary changes, and therapeutic response provided a compelling biological and public-health rationale for proposing a nationwide multivitamin supplementation program. The intervention therefore represented a population-level response to converging evidence that nutritional inadequacy—potentially interacting with other environmental and toxic exposures—played a major role in the epidemic.

The distribution of oral multivitamin supplements (including B vitamins) started with the population of Pinar del Río in March, 1993 and was extended to all the Cuban population in May 1993. A substantial reduction of disease incidence was quickly observed as of June, 1993. This reinforced the concept that nutritional status -but not a specific vitamin or nutrient- modified susceptibility to the neuropathy, as the pathological process may emerge from the interaction between inadequate nutritional reserves and environmental/toxic exposures (Centers for Disease Control and Prevention, 1994; Román, 1994b; Colectivo de Autores, 1995; The Cuba Neuropathy Field Investigation Team, 1995).

This interpretation was nevertheless contested. Critics in Cuba argued that comparable syndromes had not been reported in countries experiencing more severe nutritional deficiencies and noted that Cuba had maintained relatively high nutritional and health indicators immediately before the onset of the Special Period. Subsequently, historical accounts emerged describing a similar condition among the Cuban population during the severe deprivation associated with the nineteenth-century Wars of Independence. In this context the Spanish General Valeriano Weyler concentrated the Cuban peasants living and working in rural areas in towns, to limit their support of the Cuban independence forces, paralyzing agriculture in the country. At the same time, a Naval blockade imposed by the US Army to avoid Spanish ships from entering the island, aggravated even more the situation of the Cuban population, already greatly affected by a prolonged war. These conditions lead to the first outbreak of neuropathy in Cuba, which was excellently recognized and described between 1898 and 1900 by two Cuban physicians. These findings reinforced analyses that linked the contemporary outbreak to nutritional deficiencies (Santiesteban, 1997; Santiesteban et al., 2000). Likewise, Gustavo Román drew parallels between the epidemic and historical neuropathies associated with famine and wartime deprivation, arising under conditions of severe socioeconomic hardship and scarcity (Román, 1994a). Additionally, the historical investigations in Cuba also led to rescuing the primacy of the discovery of red free light funduscopy in 1861 by the Cuban ophthalmologist Francisco Argilagos Ginferrer. Red free light funduscopy proved to be pivotal for the demonstration of the selective loss of the papillomacular bundle which was included in the diagnostic criteria of CEN (Santiesteban Freixas, 2015).

Alternatively, a viral hypothesis was also considered during the early stages of the epidemic. This interpretation was initially supported by the apparent clustering and geographic spread of cases, and subsequently, by the identification of a virus, with typical enterovirus cytopathic effect (CPE) from a small number of patients (Más Lago et al., 1993). A pivotal moment occurred on April, 1993—sixteen months after the first cases were identified—when “a viral agent” with mild CPE was reported to have been isolated in 96% of CSF samples from affected patients. Genomic analysis revealed a 91% homology with the enterovirus Coxsackie A9 (Más et al., 1997; Rodríguez et al., 1998). This finding brought renewed prominence to the viral hypothesis, and reignited debate regarding the disease’s etiology.

Cuban neurologists and neurophthalmologists had argued from the beginning that several aspects excluded an infectious origin of the epidemic. Firstly, epidemiological studies failed to demonstrate an infectious pattern of spreading and also, the clinical features of optic and peripheral nerve impairment was not in line with an infectious etiology (Román, 1994b; Sadun et al., 2025). Neuropathological and electrophysiological findings pointed to a predominantly axonal process, more consistent with toxic–metabolic injury than with inflammatory demyelination (Román, 1994a). Additionally, early laboratory findings mostly indicated normal CSF samples, without increases in cell count. Occasional CSF abnormalities—such as mild intrathecal immunoglobulin synthesis and transient alterations in blood–CSF barrier permeability—were infrequent and insufficient to support an inflammatory or immune-mediated process (Alfaro et al., 1994; González-Quevedo Monteagudo et al., 2008). Thus, the relationship between the reported viral findings and disease pathogenesis remained uncertain.

When the PAHO and ORBIS missions arrived in May 1993, Cuban experts and health authorities briefed them on the main findings, emphasizing the possibility of a viral contribution. Dr. Daniel Carleton Gajdusek—a renowned neurovirologist from the NIH in Washington and recipient of the 1976 Nobel Prize in Medicine—received detailed information on the viral findings. Nevertheless, he quickly dismissed this hypothesis, citing the lack of correlation with clinical and cerebrospinal fluid (CSF) findings. His opinion, which aligned with the arguments advanced primarily by Cuban physicians, together with the failure to confirm viral isolations in external laboratories, ultimately consolidated the dismissal of the viral hypothesis (Román, 1994b; Román, 2016).

The 1997 update of the National Program on Epidemic Neuropathy recognized that, the condition was best characterized as an optic neuropathy of probable toxic–nutritional origin. Importantly, in that document no reference was made to viral involvement (Serrano Verdura et al., 1997).

## 3 Pathophysiological mechanisms underlying Cuban epidemic neuropathy

This multifactorial toxic–nutritional model is particularly relevant to explain the pathophysiology of CEN. Epidemiological observations linked disease risk to dietary intake of several B vitamins and other nutrients, rather than with a single nutritional variable in the absence of overt malnutrition (Sadun et al., 1994; Jiménez et al., 1995). Thiamine (B1) deficiency showed the strongest link to neuronal dysfunction (Macías-Matos et al., 1996), as it impairs mitochondrial ATP production and increases oxidative stress in energy-demanding neural tissues. Deficiencies in riboflavin and niacin amplify these effects, while folate and B12 affect one-carbon metabolism and methylation (Tucker and Hedges, 1993; Rodríguez-Hernandez et al., 2001). No single deficiency explains the full CEN spectrum, but their combined effect may reduce neural metabolic reserve.

Nutritional deficits may also have weakened antioxidant defenses. Vitamins, micronutrients, methionine derivatives, and carotenoids support redox balance and membrane protection; their depletion could increase neuronal and mitochondrial vulnerability to oxidative injury (Román, 1994b).

Methionine, an antioxidant, and taurine—abundant in neural tissue and retina—were considered relevant. Taurine supports membrane stability, calcium homeostasis, mitochondrial function, and antioxidant defense (Jakaria et al., 2019). Though not a primary cause, reduced taurine levels were noted in optic-form patients (González-Quevedo et al., 2001), suggesting it might modify neuronal vulnerability.

Toxic exposures - cyanide and methanol-derived formate - inhibit cytochrome c oxidase, blocking oxidative phosphorylation and causing neuronal injury. Detoxification involves B vitamins, folate, and sulfur amino acids like methionine, so nutritional depletion may impair detoxification. Methanol content in illicit beverages was increased (Sadun et al., 1994); while patients displayed elevated formate and reduced folate (Eells et al., 2000), and animal models of chronic low-dose methanol exposure in folate deficient rodents revealed optic nerve damage (Sadun, 1998; González-Quevedo et al., 2018). Cyanide from tobacco and dietary sources was also linked to increased risk (Román, 1994b; The Cuba Neuropathy Field Investigation Team, 1995). These otherwise insufficient toxic exposures likely acted within a multifactorial model, where poor nutrition magnified their effects.

A unifying mechanism linking these nutritional abnormalities is mitochondrial dysfunction. When oxidative phosphorylation is impaired, ATP availability decreases and mitochondrial electron leakage can increase the generation of reactive oxygen species (ROS). Consequently, mitochondrial dysfunction can simultaneously produce energy failure and oxidative injury, that ultimately result in selective neuronal death. Fine, unmyelinated fibers (papillomacular bundle) and longer fibers (peripheral nerves) are the most vulnerable, because they depend heavily on mitochondrial oxidative phosphorylation to maintain axonal membrane gradients, action potential propagation, axonal transport, and synaptic function (Sadun, 1998; Sadun, 2002; Carelli et al., 2004).

Oxidative stress may have acted as an important convergent pathway in CEN. Excess ROS can damage mitochondrial proteins, membrane lipids, DNA, and components of the electron transport chain, creating a self-reinforcing cycle in which mitochondrial dysfunction promotes further ROS production and oxidative damage further impairs mitochondrial function. Neuronal membranes are particularly susceptible because of their high content of polyunsaturated fatty acids. Oxidative damage to membranes and mitochondrial structures can impair axonal transport and ultimately trigger axonal degeneration (Sadun, 1998; Sadun, 2002; Carelli et al., 2004). The proposed pathophysiological mechanism of CEN is outlined in the Figure 1.

**FIGURE 1:**
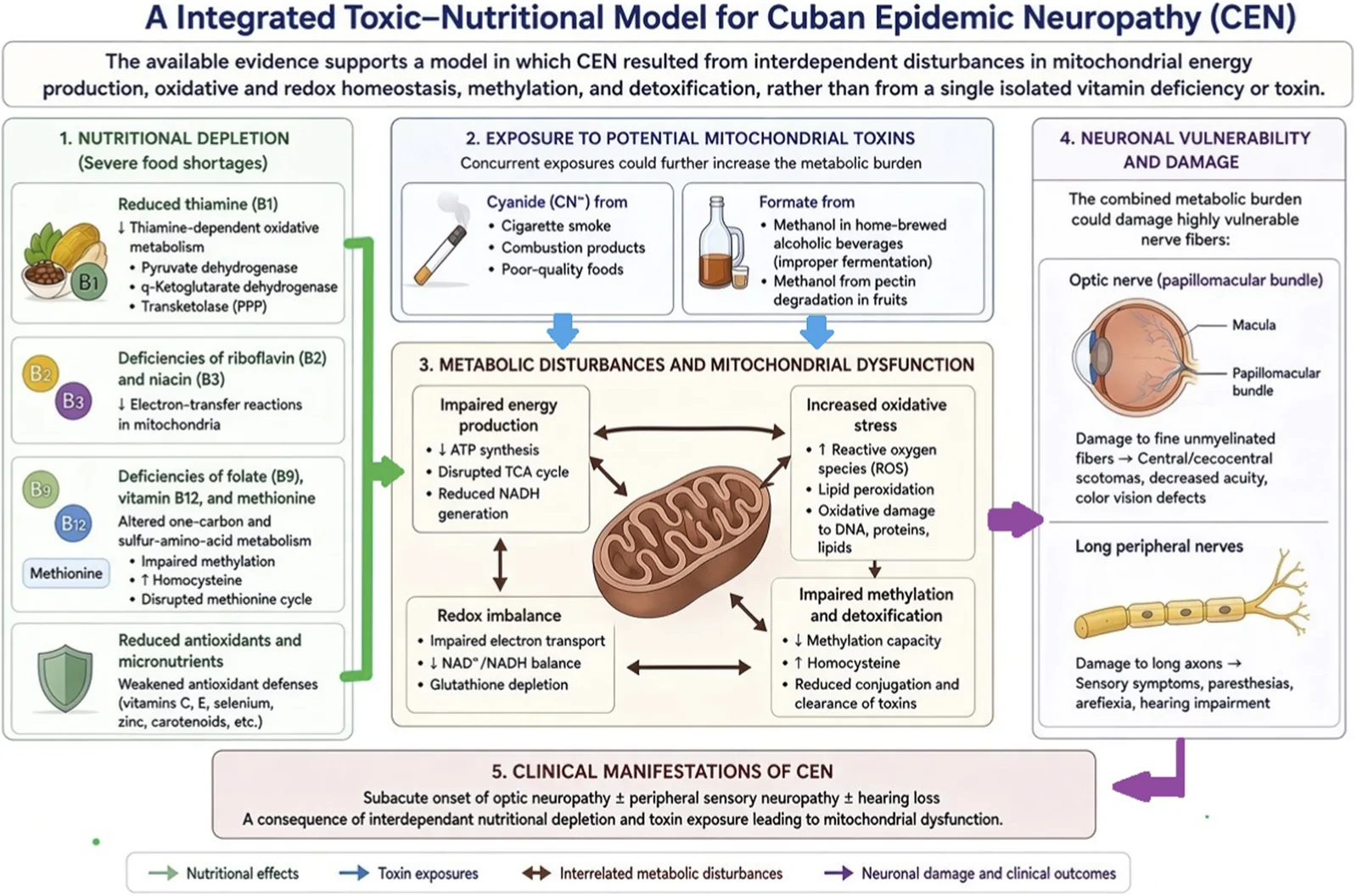
Proposed toxic–nutritional model of Cuban epidemic neuropathy (CEN). Severe nutritional depletion, including deficiencies of B vitamins, methionine, antioxidants, and micronutrients, together with potential exposure to cyanide and methanol-derived formate -from altered home-brewed alcoholic beverages and pectin degradation in fruits-, may have produced interdependent disturbances in mitochondrial energy production, redox balance, methylation, and detoxification. These convergent metabolic abnormalities promote impaired ATP generation, oxidative stress, and reduced detoxification capacity, creating a self-reinforcing cycle of mitochondrial dysfunction and oxidative injury. The resulting metabolic burden preferentially affects highly vulnerable nerve fibers, particularly the papillomacular bundle of the optic nerve and long peripheral axons, leading to the characteristic optic and peripheral neuropathic manifestations of CEN. (AI-assisted image).

## 4 Institutional dynamics and the politicization of scientific inquiry

Given the geopolitical context in which the Cuban neuropathy outbreak emerged, CEN has been described as possibly the first major epidemic of neurological disease in which political and socioeconomic conditions were central to its emergence (Román, 1994b). This context inevitably influenced the scientific debate surrounding its etiology.

The trajectory of the controversy illustrates how political and administrative decisions can shape the production and circulation of scientific knowledge during a public health crisis. Numerous reports prepared by Vice Minister Dr. Héctor Terry Molinert and his team documented epidemiological and clinical findings that strongly supported a nutritional contribution to the epidemic. However, this information apparently did not reach the highest levels of decision-making promptly, while the emerging viral hypothesis received greater attention among some authorities. This divergence may have contributed to delays in coordinated action and differences in the dissemination and public communication of scientific findings. The dismissal of Dr. Terry in April 1993 further intensified the perception that scientific and administrative considerations had become closely intertwined (Figaredo Curiel et al., 2023; Román, 2016).

The shift in emphasis had implications beyond the scientific debate. By directing attention toward a potentially infectious cause, it temporarily displaced consideration of the structural and nutritional determinants that many frontline clinicians and researchers had identified from the earliest epidemiological and clinical observations. At the time, a nutritional explanation may have been politically sensitive because it could be interpreted as evidence of systemic vulnerability or institutional failure, although the severe food shortages were also clearly linked to the economic crisis and external constraints, including the US blockade. In contrast, a viral explanation may have appeared politically less sensitive because it framed the epidemic as an external biomedical event rather than as a consequence of socioeconomic conditions (Figaredo Curiel et al., 2023).

Ultimately, the controversy was progressively resolved through the convergence of international assessments, which supported the nutritional component initially emphasized by Cuban clinicians and researchers, and the striking epidemiological response to the nationwide vitamin supplementation program. Together, these developments provided compelling evidence for a major toxic–nutritional contribution to CEN and helped redirect subsequent research toward the metabolic, mitochondrial, and environmental mechanisms underlying the epidemic.

## 5 Discussion and future perspectives

The broader legacy of CEN illustrates how intensive national and international collaboration can contribute to solving complex public health problems. The epidemic functioned as a natural laboratory in which the effects of interacting nutritional, metabolic, and environmental stressors on the nervous system could be observed at the population level. This unique circumstance provided valuable insights into mitochondrial vulnerability and helped clarify mechanisms that may also operate in other acquired and genetic neuropathies. Several optic neuropathies share clinical and pathological features with the optic form of CEN, including tobacco-alcohol amblyopia, Leber hereditary optic neuropathy (LHON), nutritional deficiencies—particularly folate and vitamin B12 deficiency—and toxic optic neuropathies caused by ethambutol, methanol, cyanide, and other agents that interfere with oxidative phosphorylation (Sadun, 1998; Sadun, 2002; Carelli et al., 2004). These overlapping phenotypes support the concept that distinct combinations of biological, nutritional, and environmental insults can converge on common pathways of mitochondrial dysfunction and axonal injury.

This framework also provides a biologically coherent interpretation of the therapeutic and epidemiological response to the epidemic. An important lesson is that early nutritional intervention may be beneficial even when the specific contributions of individual nutrients or toxins remain uncertain. The clinical improvement observed with multivitamin therapy and the rapid decline in disease incidence following nationwide supplementation are consistent with a substantial and potentially reversible metabolic component. These observations strengthen the interpretation of CEN as predominantly a toxic–nutritional/metabolic disorder, while not establishing that any single deficiency or toxin was sufficient to cause the disease. Rather, they support the hypothesis that restoring nutritional and metabolic reserve may have interrupted the pathogenic cascade before irreversible axonal degeneration developed.

CEN also provides a compelling case study of how scientific inquiry operates within, and can be constrained by, institutional and political contexts. The evolution of the etiological debate reflected not only the accumulation of empirical evidence but also changes in scientific communication, the incorporation of frontline clinical observations into research agendas, and greater openness to competing explanations. The experience underscores the importance of maintaining broad diagnostic and etiological hypotheses, transparent communication, and an evidence-based approach to public health emergencies. Scientific responses are most effective when patient-centered evidence remains the primary guide and when potentially sensitive explanations are evaluated objectively rather than being prematurely excluded or favored for reasons external to the evidence.

More than three decades later, Cuba again faces a complex economic, social, and geopolitical environment with some similarities to the circumstances of the Special Period. Although no comparable nutrition-related epidemic has emerged to date, the absence of such an outbreak should not obscure the potential health consequences of prolonged nutritional and socioeconomic stress. The slower evolution of the current crisis, together with greater access for part of the population to external income, remittances, and other forms of support, may have mitigated some of the nutritional consequences observed during the early 1990s. Nevertheless, further deterioration in food security and access to essential resources could increase vulnerability to nutritional and environmentally mediated neurological disorders. The experience of CEN therefore remains relevant not only as a historical episode but also as a reminder that economic and political conditions can become important determinants of population health, and that prevention requires early recognition of nutritional vulnerability and timely, evidence-based intervention.

## Data Availability

The original contributions presented in the study are included in the article/supplementary material, further inquiries can be directed to the corresponding author.
